# Aperiodic EEG signatures: unveiling the interplay between APOE ε4 and mild cognitive impairment subtypes

**DOI:** 10.3389/fnagi.2025.1675330

**Published:** 2026-01-08

**Authors:** Joel Eyamu, Boncho Ku, Kahye Kim, Kun Ho Lee, Jaeuk U. Kim

**Affiliations:** 1 Division of Digital Health Research, Korea Institute of Oriental Medicine, Daejeon, Republic of Korea; 2 KM Convergence Science, University of Science and Technology, Daejeon, Republic of Korea; 3 Gwangju Alzheimer’s and Related Dementia (GARD) Cohort Research Center, Chosun University, Gwangju, Republic of Korea; 4 Department of Biomedical Science, Chosun University, Gwangju, Republic of Korea; 5 Dementia Research Group, Korea Brain Research Institute, Daegu, Republic of Korea

**Keywords:** mild cognitive impairment, Alzheimer’s disease, electroencephalography, APOE ε4, aperiodic component, parameterized spectral estimation, periodic component

## Abstract

**Background:**

Mild cognitive impairment (MCI) is a cognitive decline syndrome in the elderly, often a precursor to dementia. It is a heterogeneous condition that can signal degenerative disorders like Alzheimer’s or non-degenerative conditions such as vascular issues, depression, or poorly managed diabetes. Early detection of MCI is crucial for timely intervention, and differentiating its phenotypes helps in understanding its causes, progression, and treatment. EEG, which records brain electrical activity, consists of rhythmic and arrhythmic components. Examining these inherently overlapping EEG components calls for quantification, ensuring that an appropriate physiological mechanism is attributed to a given neural response. This study explores the interaction between APOE ε4 (APOE4) and cognitive impairment on non-oscillatory EEG activity.

**Methods:**

We examined aperiodic EEG activity using a parameterized spectral estimation approach in a sample comprising 751, 142, and 279 cognitively normal (CN), non-amnestic (naMCI), and amnestic (aMCI) MCI patients, respectively. The 5-min EEG was recorded using a prefrontal two-channel EEG device in a resting state, eyes closed. Cognitive decline was assessed using the Seoul Neuropsychological Screening Battery (SNSB) and the Mini-Mental State Examination (MMSE). The analyses were performed using various statistical methods, including independent *t*-tests and generalized linear models (GLM) with an identity link function. These analyses investigated the main and interaction effects of the APOE4 status and participants’ cognitive states.

**Results:**

We found interactions between APOE4 and cognitive states in the aperiodic EEG exponent and the spectral power ratio (SPR). Distinct patterns were observed in the exponent, offset, and SPR between APOE4 non-carriers and carriers across the CN, naMCI, and aMCI. Among the APOE4 carriers, the aMCI individuals exhibited heightened aperiodic activity and a reduced SPR than the naMCI. Furthermore, the CN had a lower SPR compared to the naMCI. However, no differences in the aperiodic component and SPR were observed in the APOE4 non-carriers across the cognitive states.

**Discussion:**

The higher aperiodic component and a reduced SPR observed in aMCI relative to naMCI in APOE4 carriers may indicate an interplay between genetic predisposition, neuropathological changes, and cognitive decline. These aperiodic components, combined with APOE4 status, represent promising neurophysiological markers that may help identify individuals at elevated risk for cognitive decline or progression toward AD.

## 1 Introduction

Mild cognitive impairment (MCI), estimated to affect 15%–20% of persons aged 60 and above, is a syndrome characterized by cognitive decline that exceeds a typical person’s age and education level but doesn’t disrupt their activities of daily living (Petersen, 2016). MCI could be an initial stage for several degenerative, vascular, psychiatric, and medical disorders, potentially advancing to degenerative conditions such as Alzheimer’s disease (AD) dementia, frontotemporal dementia (FTD), and dementia with Lewy bodies (DLB). Moreover, it may be a symptom in non-degenerative conditions such as vascular cognitive impairment (VCI), major depressive disorder, generalized anxiety disorders, uncompensated heart failure, and poorly managed diabetes mellitus (Petersen, 2016). MCI can be classified into amnestic MCI (aMCI) and non-amnestic MCI (naMCI) (Petersen, 2004). The aMCI is primarily characterized by memory impairment, whereas naMCI involves deficits in other cognitive domains, such as attention, executive function, visuospatial skills, and language; both conditions, however, can impact one or multiple neuropsychological domains (Petersen, 2004; Dugger et al., 2015). The number of affected domains is crucial in assessing the magnitude of underlying brain pathology, the disease’s impact, and the probability of transitioning to dementia (Petersen, 2016). Of the MCI subtypes, the aMCI is the most likely to convert to AD dementia (Yaffe et al., 2006); and therefore, regarded as a precursor to AD dementia (Kim et al., 2022).

For therapeutic interventions, early detection of MCI before conversion to AD is crucial for patient management (Kim et al., 2022). Moreover, distinguishing MCI by phenotypes, i.e., aMCI and naMCI, may provide insights into underlying etiology, pathophysiology, and prognosis, ultimately providing vital information for treatment approaches and strategies. Important in the approach to understanding cognitive impairment is the study of its underlying modifiable and non-modifiable factors, such as age, or even APOE ε4 carrier genotype, a known risk factor of AD, associated with brain alpha rhythm slowing and functional network alterations in normal aging (Ponomareva et al., 2022). Studies such as (Oveisgharan et al., 2018; Gharbi-Meliani et al., 2021) have associated APOE ε4+ phenotype with MCI/AD and its pathology, as well as accelerated cognitive decline in APOE4 carriers compared to non-carriers (Polsinelli et al., 2023). In a review by Miyashita et al. (2023) APOE genotypes play a crucial role in lipid metabolism and subsequently AD pathology, with APOE4 posing a genetic risk of AD while APOE e2 plays a protective role. Moreover, the APOE ε4 allele confers a roughly 4-fold higher risk in clinically diagnosed individuals, increasing to 6-fold in neuropathologically confirmed cases (Reiman et al., 2020).

Electroencephalogram (EEG), a recording of brain electrical activity, has emerged as a promising, non-invasive, and cost-effective method for screening and tracking AD and MCI (Lee et al., 2025; Yuan and Zhao, 2025). EEG consists of the periodic activity and the arrhythmic or background activity, otherwise known as the aperiodic activity. The previous studies that calculated the averaged power in predefined frequency bands notably fail to address the potential confounding of the aperiodic neural activity (Donoghue et al., 2020). This inherent overlap between these EEG components calls for quantification that separates the signal to ensure an appropriate physiological mechanism is attributed to a given neural response (Ostlund et al., 2022). For instance, some studies removed aperiodic activity from analyses to emphasize brain oscillations as they considered it as neural noise (He, 2014).

Recently, aperiodic components have garnered considerable attention, as they are not merely unstructured noise but hold distinct functional significance (Tröndle et al., 2023). This EEG power spectrum component is thought to arise from asynchronous spiking and postsynaptic potentials in neuronal populations (Donoghue et al., 2020; Ouyang et al., 2020). Although recent studies highlight considerable variability, presenting an ongoing debate regarding its physiological interpretation and biological significance (Waschke et al., 2021; Tröndle et al., 2022, 2023), it has nonetheless been proposed as a non-invasive proxy for neural excitation-inhibition (E/I) dynamics (Gao et al., 2017; Donoghue et al., 2020). The aperiodic activity contributes power across all the representative frequencies and is described by an exponential decrease in power across increasing frequencies following a 1/f distribution. This 1/f behavior is quantified using the exponent (slope), which describes the pattern of power across frequencies and the aperiodic offset**—**a broadband offset of the spectrum (Donoghue et al., 2020; Ostlund et al., 2022; Cesnaite et al., 2023), which represents a uniform shift in power across frequencies (Donoghue et al., 2020) and the overall neuronal spiking rates (Miller et al., 2014). Emerging evidence suggests that aperiodic activity may provide distinct and complementary information about brain function and pathology in neurodegenerative diseases (Azami et al., 2023; Chu et al., 2023; Kopčanová et al., 2024; Wang et al., 2024), personality (Pacheco et al., 2024), sleep (Rosenblum et al., 2024), aging (McSweeney et al., 2023; Smith et al., 2023; Montemurro et al., 2024), and cognitive speed (Ouyang et al., 2020; Azami et al., 2023; Chu et al., 2023; Kopčanová et al., 2024; Wang et al., 2024). In the context of MCI/AD research, (Azami et al., 2023) did not find any differences in the aperiodic component between AD, MCI, and cognitively normal (CN) individuals while some studies identify robust aperiodic shifts in AD i.e., elevated offsets and exponents (Chu et al., 2023). Steeper aperiodic slopes in AD have been associated with increased inhibition, contrasting the evidence of amyloid-driven local hyperexcitability, highlighting a potential disconnect between micro-scale pathological effects (Wang et al., 2024). Notably, the specificity of aperiodic changes across neurodegenerative diseases remains unclear, raising concerns about their reliability as standalone diagnostic markers. Moreover, few studies have explored aperiodic shifts across different MCI phenotypes.

This study aimed to investigate how the APOE ε4 allele and MCI phenotypes jointly influence the aperiodic component of EEG spectral power, an interplay that has not been previously explored. We hypothesize that APOE ε4 modulates the differences in non-oscillatory neural activity between aMCI and naMCI. Our study revealed a heightened aperiodic component in the aMCI compared to the naMCI group, with these differences being particularly pronounced among APOE ε4 carriers. Furthermore, older MCI individuals (≥70 years) showed reduced periodic measures of center frequency, peak power, and peak bandwidth compared to the early MCI group (<70 years), a pattern most evident in the naMCI group. In contrast, age-related distinctions in aperiodic components were not apparent across the MCI subgroups.

The distinct EEG spectral aperiodic components between aMCI and naMCI, considering APOE4 status, could serve as a potential marker for identifying individuals at higher risk of cognitive decline or progression to AD.

## 2 Materials and methods

### 2.1 Participants

**FIGURE 1:**
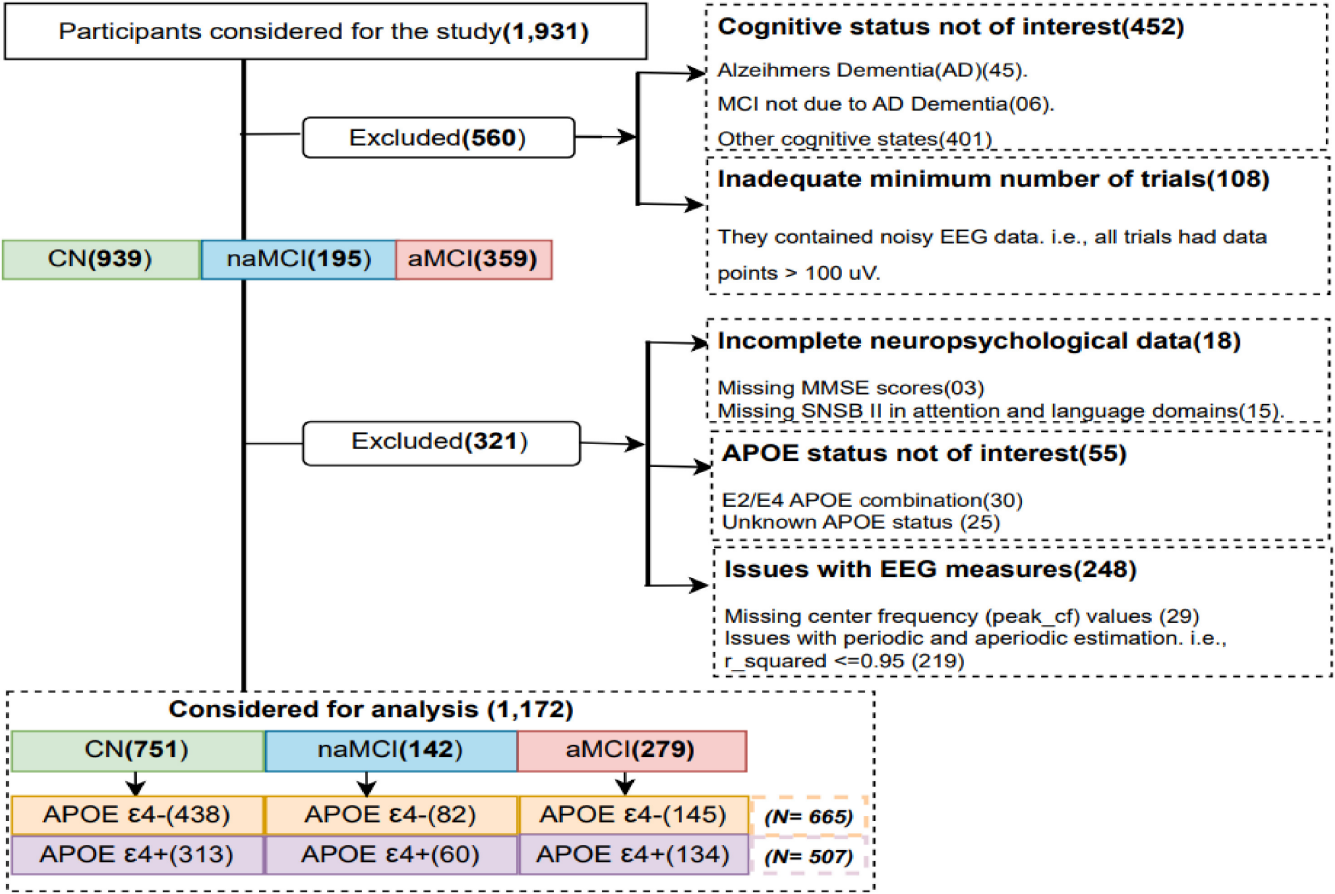
Participant inclusion and exclusion process; CN, cognitively normal; naMCI, non-amnestic mild cognitive impairment; aMCI, amnestic mild cognitive impairment.

This work involved 1,931 participants recruited between October 2018 and December 2022 at the Gwangju Alzheimer’s Disease and Related Dementia (GARD) center (Gwangju City, South Korea). A total of 881 participants were excluded from the analysis due to several reasons, such as cognitive status not of interest (*n* = 452), possessing extremely noisy data (*n* = 108), had incomplete neuropsychological information (*n* = 18), APOE4 status not of interest (*n* = 55), and issues with the extracted EEG measures such as missing values (*n* = 29). Furthermore, all the periodic and aperiodic estimation models that did not meet the condition of R^2^ ≥ 0.95 were not considered (*n* = 219) (Wang Z. et al., 2022) (See Figure 1).

The participants considered in this study were examined through a clinical interview, which included assessment of the clinical dementia rating (CDR), and met the Petersen criteria (Petersen, 2004) and had a CDR score of 0.5. Additionally, their neuropsychological test z scores were below −1.5 on at least one of the five domain tests according to age, education, and sex-specific norms (Opwonya et al., 2022). The MCI groups were then divided into aMCI, if the memory cognitive domain was primarily affected, and naMCI, if other cognitive domains were affected (Petersen, 2004).

For final analysis, the CN participants were 751 (334 men and 417 women) with a mean age ± standard deviation of 72.30 ± 6.04, patients with naMCI were 142 (59 men and 83 women), with mean age ± standard deviation of 72.57 ± 6.12 years and those with aMCI were 279 participants (158 men and 121 women), with mean age ± standard deviation of 73.74 ± 6.47 years (Table 1). Furthermore, the participants were stratified into <70, ≤75, and >75 years, based on previous results on the same participants that showed the emergence of differences in MCI EEG patterns from 70 years (Eyamu et al., 2025).

**TABLE 1:** Demographic and neuropsychological characteristics based on the participant cognitive status.

Characteristic	CN, *N* = 751^1^	naMCI, *N* = 142^1^	aMCI, *N* = 279^1^	T-statistic	*P*-value^2^
**Demographic characteristics and APOE4**					
Age	72.30 (6.04)	72.57 (6.12)	73.74 (6.47)	12.836	**0.002**
Sex				14.005	**<0.001**
Female	417/751 (56%)	83/142 (58%)	121/279 (43%)	–	–
Male	334/751 (44%)	59/142 (42%)	158/279 (57%)	–	–
EDUYR	11.07 (4.29)	11.35 (4.23)	11.08 (4.57)	0.544	0.8
APOE4				3.409	0.2
Negative	438/751 (58%)	82/142 (58%)	145/279 (52%)	–	–
Positive	313/751 (42%)	60/142 (42%)	134/279 (48%)	–	–
**Neuropsychological test scores**					
MMSE	27.82 (1.76)	26.82 (2.45)	26.32 (2.69)	80.837	**<0.001**
Attention	9.70 (2.14)	8.38 (2.07)	8.62 (1.91)	83.063	**<0.001**
Language	0.20 (0.27)	−0.11 (0.45)	−0.07 (0.46)	143.178	**<0.001**
Visuospatial	0.50 (0.39)	0.23 (0.71)	0.17 (0.77)	53.016	**<0.001**
Memory	0.27 (0.57)	0.02 (0.52)	−0.71 (0.54)	405.671	**<0.001**
Frontal	0.25 (0.55)	−0.17 (0.65)	−0.23 (0.70)	119.885	**<0.001**
**EEG measures**					
exponent	0.98 (0.35)	0.97 (0.33)	1.00 (0.37)	0.214	0.9
offset	1.14 (0.40)	1.13 (0.37)	1.17 (0.41)	0.685	0.7
peak_cf	9.33 (0.84)	9.32 (0.76)	9.22 (0.89)	2.442	0.3
peak_pw	0.68 (0.35)	0.71 (0.33)	0.67 (0.35)	2.095	0.4
peak_bw	2.41 (0.60)	2.44 (0.51)	2.41 (0.58)	3.531	0.2
SPR	0.77 (0.48)	0.81 (0.47)	0.72 (0.43)	3.871	0.14

^1^Mean (SD); *n*/*N* (%).

^2^Kruskal-Wallis rank sum test; Pearson’s Chi-squared test. The study comprised of 751 CN, 142 naMCI, and 279 aMCI individuals. There was a significant difference in the gender distribution in the two groups (*p* < 0.001). The CN group consisted of 56% women and 44 % men, and naMCI group consisted of 58% women and 42% men, while the aMCI group had 43% women and 57% men. *Post hoc* comparisons for the significant features are in Supplementary Table 1. Significant variables (value of *p*≤0.05) are bolded.

### 2.2 Neuropsychological battery

The participants underwent cognitive function assessment using the latest version of the SNSB (SNSB II) (Kang et al., 1997, 2003). The Korean Mini-Mental State Examination (K-MMSE) was also employed within this cognitive function assessment as the primary screening tool.

### 2.3 Apolipoprotein E genotyping

The APOE genotype was determined in all participants from genomic DNA from leukocytes in whole blood using the QuickGene DNA kit S from KURABO Inc., (Osaka, Japan) (Kang et al., 2024).

The participants were grouped into APOE4 carriers (ε3/ε4 or ε4/ε4) and APOE4 non-carriers (ε3/ε3 or ε2/ε3). Because of the potential protective effect of APOE ε2 allele (Suri et al., 2013), the subjects with two of its copies were excluded in this analysis.

### 2.4 EEG recording

Electroencephalogram was recorded using NeuroNicle FX2 (LAXTHA, Daejeon, South Korea) device from two monopolar scalp electrodes placed in Fp1 and Fp2 with a reference on the right earlobe based on the international 10–20 system. Despite its limitations to recording neural activity only from the prefrontal cortex, this portable, dry electrode device, optimized for clinical settings, offers a practical balance between signal quality and electrode preparation time (Choi et al., 2020). The frequency band-pass of the amplifiers was 3–43 Hz, and the input range was +/−393 μV (Input noise < 0.6 μVrms). Electrode contact impedances were maintained below 10 kΩ, and data were digitized at a sampling frequency of 250 Hz and 15-bit resolution. All filters were digital, and IIR Butterworth filters were applied. Band stop: 2nd order with f1 = 55 Hz and f2 = 65 Hz. High-pass filter: 1st order with fc = 2.6 Hz. Low-pass filter: 8th order with fc = 43 Hz (Choi et al., 2019; Doan et al., 2021).

Participants were required and instructed to sit comfortably with their eyes closed, while qualified operators watched both participant sleepiness and EEG traces to minimize artifacts from muscle and eye movements. Verbal instructions before and during the recording were issued to ensure alertness (Eyamu et al., 2023). The EEG signals were acquired in a sequence of conditions of 5 min of resting state, 8 min of sensory-evoked potentials, and 5 min of selective attention tasks. To keep a controlled environment during data collection, the experiments were conducted in a quiet room with standardized illumination. Furthermore, based on the participant’s visit schedule, the EEG was recorded at varying times of the day between 9:00 a.m. and 5:00 p.m.

Further details of the EEG experiment(s) can be found in the studies (Choi et al., 2019; Doan et al., 2021; Eyamu et al., 2023, 2024). For this study, the EEG collected under the resting state eyes closed condition was considered for analysis.

### 2.5 Data pre-processing

**FIGURE 2:**
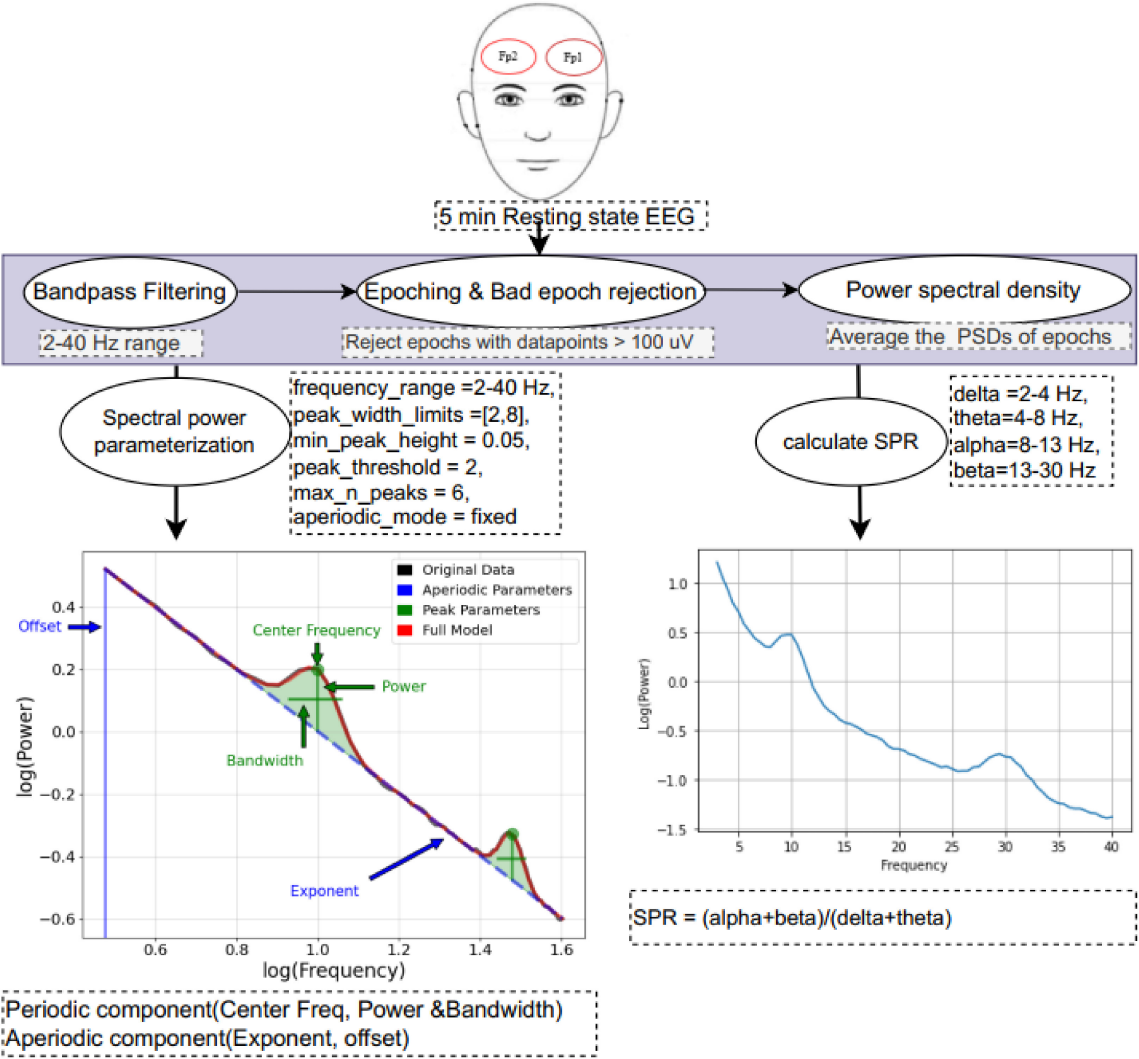
Electroencephalogram (EEG) data preprocessing and feature extraction workflow: 5 min of resting-state EEG were recorded under the eyes-closed condition. Offline preprocessing included band-pass filtering (2–40 Hz), segmentation into non-overlapping 2-s epochs, and exclusion of epochs with amplitudes exceeding ±100 μV. Power spectral density (PSD) for each epoch was estimated using Welch’s method [MNE-Python’s psd_array_welch (Gramfort et al., 2013)], and the final power spectral density (PSD) was computed by averaging across epochs. The specparam tool (Donoghue et al., 2020) was then applied to decompose the PSD into aperiodic and periodic components. Additionally, the spectral power ratio (SPR) was calculated using conventional spectral power analysis.

The EEG was prepared and processed using custom scripts written in Python (version 3.8.16). The features extracted for the present study are illustrated in Figure 2. The data was segmented into 2 s non-overlapping epochs. Subsequently, all epochs with data points above±100 μV were eliminated (Eyamu et al., 2024).

### 2.6 Data analysis

The power spectral density (PSD) estimation for each of the epochs was performed using Welch from MNE python’s *psd_array_welch* method (Gramfort et al., 2013) with a 1 s Hamming window, no overlap, and a 0.5 Hz frequency resolution. The ensuing PSD used in fitting by specparam (Donoghue et al., 2020) to obtain separate periodic and aperiodic components was obtained by averaging over each epoch’s PSD (see Figure 2 and Supplementary Figure 1 for a sample 20 participants in the study). The goodness of fit was estimated by comparing each fit with the original PSD in terms of the mean absolute error and the R^2^ of the fit. The estimation parameters were set as; peak_width_limits = 2–8, min_peak_height = 0.05, peak_threshold = 2.0, max_n_peaks = 6, the fitted frequency range = 3–40 Hz and aperiodic_mode = “fixed.”

Our analysis involved band-limited power of fitted spectra of the periodic components in the dominant alpha band (5–13 Hz) (Choi et al., 2019). Furthermore, we calculated the spectral power ratio (SPR) (Benwell et al., 2020a; Flores-Sandoval et al., 2023) based on the conventional band-based spectral analysis approach.

### 2.7 Statistical analysis

The statistical analyses were carried out using R Studio (version 2022.07.2+576), running on R (version 4.1.3) for Windows, including packages gtsummary (version 1.6.1), ggplot2 (version 3.4.0) and corrplot (version 0.92) (R Core Team, 2022; Sjoberg et al., 2021; Wei and Simko, 2021; Wickham, 2016) with a significance level of α = 0.05 for all tests. Independent sample *t*-tests were performed using Kruskal-Wallis rank sum test for continuous variables, while chi-squared tests were used for categorical variables. The false discovery rate (FDR) (Benjamini and Hochberg, 1995) correction was applied to all group-level contrasts in which multiple group comparisons were involved.

To understand the simultaneous effect of APOE4 and the cognitive state on the periodic and aperiodic components, a generalized linear model (GLM) with an identity link function was used as (see Equation 1),


$$y={\mathrm{APOE4}}_{\mathrm{Status}}*{\mathrm{Cognitive}}_{\mathrm{Status}}+\mathrm{AGE}+\mathrm{SEX}+\mathrm{EDUYR}$$ (1)

where *y* is a representative parameterized EEG measure, e.g., slope, offset, center frequency, etc., *APOE4*_*Status*_ (carrier or non-carrier), and *Cognitive*_*Status*_ (CN, aMCI, or naMCI). This analysis was controlled for the effect of age, sex, and years of education (EDUYR).

Furthermore, we examined age-related differences in EEG measures in relation to APOE genotype and cognitive state (see Equation 2). Participants were stratified into three age groups based on tertiles of the age distribution. We established thresholds at the 33*^rd^* and 66*^th^* percentiles, resulting in individuals less than 70 years (<70), between 70 and 75 years (≤75), and those above 75 years (>75). This ensured an approximately equal distribution of participants across the three groups, facilitating a balanced comparison of EEG measures across distinct age strata in the context of APOE4 genotype and cognitive status. This was captured using the following formula:


$$y={\mathrm{APOE4}}_{\mathrm{Status}}*{\mathrm{AGE}}_{\mathrm{Group}}*{\mathrm{Cognitive}}_{\mathrm{Status}}+\mathrm{SEX}+\mathrm{EDUYR}$$ (2)

where *AGE*_*Group*_ (<70, ≤75, and >75). This analysis was controlled for sex and years of education (EDUYR).

All the post hoc pairwise contrasts were corrected for multiple comparisons using the false discovery rate (FDR) (Storey, 2010). Furthermore, Cohen’s d effect sizes for the pairwise contrasts were calculated as standardized mean differences by dividing the estimated marginal mean difference (δ) by the residual standard deviation from the fitted regression models (Myers et al., 2013) (see Equation 3). Succinctly, for each pairwise contrast, Cohen’s d was computed using the formula:


$$C\,o\,h\,e\,n^{\prime }\,s\,d=\frac{E\,s\,t\,i\,m\,a\,t\,e\,d\,m\,e\,a\,n\,d\,i\,f\,f\,e\,r\,e\,n\,c\,e\,s}{R\,e\,i\,d\,u\,a\,l\,s\,t\,a\,n\,d\,a\,r\,d\,d\,e\,v\,i\,a\,t\,i\,o\,n}$$ (3)

## 3 Results

### 3.1 Participant characteristics

The patients with aMCI were the oldest, with a mean age (standard deviation) of 73.74 (6.47) years followed by naMCI patients with 72.57 (6.12) years, with CN 72.30 (6.04) years, youngest (*p* = 0.002). Furthermore, significant differences were observed in MMSE scores across groups with CN having the highest mean score (27.82 ± 1.76), followed by naMCI (26.82 ± 2.45) and aMCI (26.32 ± 2.69) (*p* < 0.001). In the SNSB domain scores, attention performance differed significantly across groups, with mean scores of 9.70 ± 2.14 in the CN group, 8.38 ± 2.07 in the naMCI group, and 8.62 ± 1.91 in the aMCI group (*p* < 0.001). Similarly, significant differences were observed in the language domain, where the CN group scored highest (0.20 ± 0.27), compared to −0.11 ± 0.45 in naMCI and −0.07 ± 0.46 in aMCI (*p* < 0.001). In the visuospatial domain, the CN group again demonstrated superior performance (0.50 ± 0.39), followed by naMCI (0.23 ± 0.71) and aMCI (0.17 ± 0.77) (*p* < 0.001). Memory scores also showed significant differences, with the CN group scoring highest (0.27 ± 0.57), followed by naMCI (−0.02 ± 0.52) and aMCI (−0.71 ± 0.54) (*p* < 0.001). Finally, frontal/executive function scores varied significantly among groups, with mean scores of 0.25 ± 0.55 for CN, −0.17 ± 0.65 for naMCI, and −0.23 ± 0.70 for aMCI (*p* < 0.001). No statistically significant differences were noted in the years of education and APOE ε4 status among the groups (Table 1). For differences in demographic and neuropsychological characteristics between APOE4 groups in each of the cognitive states, see Supplementary Table 3. Further details about these is beyond the scope of this work.

### 3.2 The relationship between APOE ε4, cognitive state and the spectral measures

**FIGURE 3:**
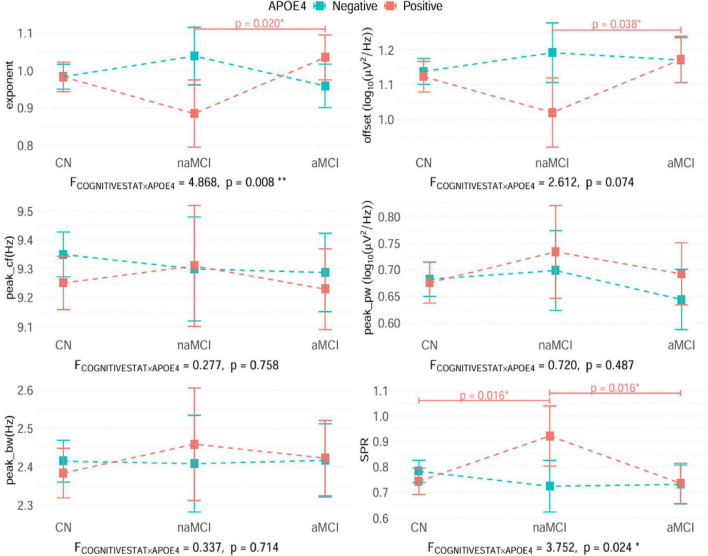
The interaction plot of the cognitive status and APOE4 in terms of the electroencephalogram (EEG) spectral measures, determined using generalized linear model (GLM) models. The interaction between APOE4 and cognitive status was tested using the ANOVA of each GLM with an identity function. F-statistics and *p*-values are notated bottom of each panel. The squares shown in the plot indicate the estimated marginal means of EEG spectral measures adjusted for sex, age, and years of education. The error bars across the squares indicate the 95% confidence intervals of the estimated marginal means. The pairwise comparisons across the cognitive status within the APOE4 groups were performed, and the obtained *p*-values are adjusted using the false discovery rate (FDR) method. Only *p*-values less than the significance level (α = 0.05) were displayed in the plot. Significance levels for the *p*-values are denoted as follows: ***p < 0.001, **p < 0.01, *p < 0.05. Exact statistical values of the models and contrasts among the three cognitive groups in each APOE4 status are presented in Supplementary Tables 4, 5, respectively. See Supplementary Figure 2 for the results of all participants, irrespective of the specparam model fit. Also see the Supplementary Table 2 for the group differences between excluded participants due to poor specParam algorithm fit (R squared < 0.95) and those considered in the main analysis (R squared ≥ 0.95).

The section below describes the results of the regression analyses to examine the relationships between various EEG measures, the APOE4, and cognitive state, adjusted for demographic characteristics of age, sex and years of education (See Figure 3). The contrasts in cognitive states by APOE4, where we determine the mean differences (*δ)* between the groups in these spectral measures are in Supplementary Table 5. No significant main effects were observed in the APOE4 and cognitive states on these parameters.

#### 3.2.1 Aperiodic parameters

We first examined whether aperiodic EEG component differed between MCI phenotypes (aMCI vs. naMCI) and were influenced by APOE4 carrier status, as these relationships were central to our study’s hypotheses.

##### 3.2.1.1 Exponent

There was a significant interaction between APOE4 and cognitive status [F_(2, 1163)_ = 4.87, *p* = 0.008]. *Post hoc* analyses revealed that among APOE4 carriers, the exponent was significantly lower in naMCI compared to aMCI [δ = −0.15, t(1163) = −2.726, Cohen’s *d* = −0.424, 95% CI: −0.28 to −0.02, *p* = 0.020], indicating a distinct aperiodic profile between these subtypes. A trend toward higher exponent values was also seen in the CN compared to naMCI [δ = 0.10, t(1163) = 1.967, Cohen’s *d* = 0.278, 95% CI: −0.02 to 0.22, *p* = 0.074]. No significant group differences were found among APOE4 non-carriers.

##### 3.2.1.2 Offset

A trend-level interaction between APOE4 status and cognitive group was observed [F_(2, 1163)_ = 2.612, *p* = 0.074]. Among APOE4 carriers, the offset was lower in naMCI compared to aMCI [δ = −0.15, t(1163) = −2.5, Cohen’s *d* = −0.389, 95% CI: −0.30 to −0.01, *p* = 0.038], with a trend toward higher offset in the CN compared to naMCI [δ = 0.10, t(1163) = 1.859, Cohen’s *d* = 0.262, 95% CI: −0.03 to 0.23, *p* = 0.095]. Sex emerged as a significant covariate [F_(1, 1163)_ = 11.745, *p* = 0.001]. No significant differences were found among non-carriers.

In summary, aperiodic EEG measures distinguished aMCI from naMCI primarily among APOE4 carriers, supporting the hypothesis that APOE4 status modulates the relationship between MCI phenotype and aperiodic neural activity.

#### 3.2.2 Periodic parameters

To assess whether periodic EEG measures varied according to MCI phenotype or APOE4 status, we analyzed peak center frequency (peak_cf), peak power (peak_pw), and peak bandwidth (peak_bw) across groups. No interactions were observed between APOE4 status and cognitive states for any of the periodic EEG measures. However, several covariates showed significant associations: age and sex were both significantly related to peak_cf [age: F_(1, 1163)_ = 30.474, *p* < 0.001; sex: F_(1, 1163)_ = 8.497, *p* = 0.004], age and sex influenced peak_pw [age: F_(1, 1163)_ = 3.941, *p* = 0.047; sex: F_(1, 1163)_ = 6.269, *p* = 0.012], and sex was significantly associated with peak_bw [F_(1, 1163)_ = 12.903, *p* < 0.001].

Summarily, the periodic EEG measures did not differ significantly by MCI subtype or APOE4 status but were influenced by demographic factors such as age and sex.

#### 3.2.3 Spectral power ratio (SPR)

To further explore EEG spectral characteristics, we examined the SPR, which reflects the balance between higher (alpha, beta) and lower (delta, theta) frequency bands and has been linked to cognitive function and dementia severity (Benwell et al., 2020b; Flores-Sandoval et al., 2023) .

There was a significant interaction between APOE4 status and cognitive group [F_(2, 1163)_ = 3.75, *p* = 0.024]. FDR-corrected post hoc analyses revealed that, among APOE4 carriers, SPR was significantly higher in naMCI compared to aMCI [δ = 0.19, t(1163) = 2.56, Cohen’s *d* = 0.400, 95% CI: 0.01–0.36, *p* = 0.016) and lower in CN compared to naMCI [δ = −0.18, t(1163) = −2.715, Cohen’s *d* = −0.382, 95% CI: −0.34 to −0.02, *p* = 0.016]. No significant differences were observed among APOE4 non-carriers across the cognitive states. Age emerged as a significant covariate [F_(1, 1163)_ = 11.62, *p* = 0.001], while sex and education were not significant contributors.

In summary, the SPR distinguished naMCI from both aMCI and controls specifically in APOE4 carriers, suggesting that the interaction between genetic risk and MCI phenotype is reflected in the spectral distribution of EEG power.

### 3.3 The relationship between APOE4, aging and the spectral measures

**FIGURE 4:**
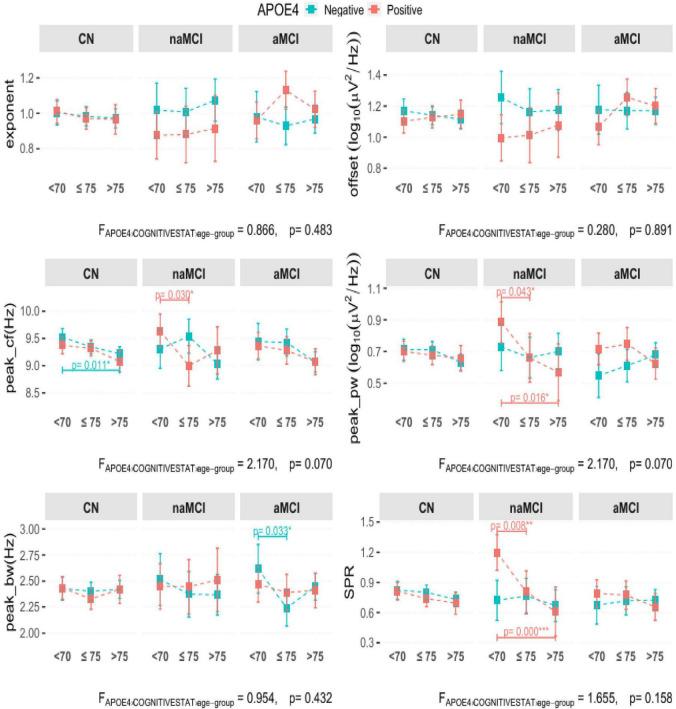
The relationship between various electroencephalogram (EEG) measures depending on APOE4 and Age group in cognitively normal (CN) and patients with mild cognitive impairment (MCI), performed using generalized linear models (GLM) with an identity link function with APOE4 and age group as factors. The age groups were divided into <70, ≤75, and >75 years. The models were adjusted for sex and years of education. *P*-values for the contrasts are adjusted using the false discovery rate (FDR) method. The black and red *p*-values or lines represent differences in APOE4 non-carriers (negative) and carriers (positive), respectively. Only significant values are shown. Significance levels for the *p*-values are denoted as follows: ***p < 0.001, **p < 0.01, *p≤0.05. Detailed statistical values are in Supplementary Tables 6, 7. See Supplementary Figure 3 for the results of all participants, irrespective of the specparam model fit.

Figure 4 presents the GLM models for various EEG parameters. Further detailed information is supported in Supplementary Tables 4, 5. These models examine the effects of APOE4, cognitive state, and the age group on various resting EEG parameters in various cognitive states while controlling for sex and years of education.

#### 3.3.1 Aperiodic parameters

To determine whether aperiodic EEG measures varied by cognitive status, APOE4 status, or age, we analyzed the exponent and offset measures across age groups. No significant main effects or interactions were observed for either the exponent or offset across cognitive groups, APOE4 status, age group, or their combinations. Sex emerged as a significant covariate for the offset [F_(1, 1152)_ = 12.327, *p* < 0.001].

#### 3.3.2 Periodic parameters

We assessed whether periodic EEG measures of peak center frequency (peak_cf), power (peak_pw), and bandwidth (peak_bw) varied according to age group, APOE4 status, and cognitive phenotype. There were no significant main effects or interactions for peak_bw and peak_pw.

##### 3.3.2.1 Peak center frequency (peak_cf)

A significant main effect in the age group was observed for peak_cf [F_(2, 1152)_ = 4.248, *p* = 0.015], no significant effects of APOE4 or cognitive status were observed, while sex was a significant covariate [F_(1, 1152)_ = 9.695, *p* = 0.002]. Furthermore, post-hoc contrasts showed that in the patients with naMCI, APOE4 carriers exhibited heightened peak frequencies in the <70 compared to the ≤75 age group [δ = 0.64, t(1152) = 2.585, Cohen’s *d* = 0.775, 95% CI: 0.05–1.23, *p* = 0.03]. Those with aMCI showed non-significant differences in both APOE4 groups across all age groups. For reference, in the CN, only APOE4 non-carriers demonstrated significantly higher peak frequencies in the <70 compared to the >75 age group [δ = 0.30, t(1152) = 2.914, Cohen’s *d* = 0.366, 95% CI: 0.05–0.55, *p* = 0.011].

##### 3.3.2 2 Peak power (peak_pw)

Group contrasts in the naMCI APOE4 carriers demonstrated significantly more peak power in <70 compared to the ≤75 (δ = 0.22, Cohen’s *d* = 0.657, 95% CI: −0.02 to 0.47, *p* = 0.043) and >75 (δ = 0.32, Cohen’s *d* = 0.923, 95% CI: 0.04–0.459 *p* = 0.016) groups. No significant differences were observed for APOE4 non-carriers across all contrasts (*p* > 0.05). Sex was a significant covariate [F_(1, 1152)_ = 5.730, *p* = 0.017].

##### 3.3.2.3 Peak bandwidth (peak_bw)

Group contrasts showed a heightened bandwidth in the <70 compared to the ≤75 age group exclusively in the aMCI APOE4 non-carriers [δ = 0.38, t(1152) = 2.544, Cohen’s *d* = 0.651, 95% CI: 0.02–0.73, *p* = 0.033].

Summarily, the periodic EEG measures, particularly peak center frequency and peak power, demonstrated age-related declines that were most evident in specific subgroups defined by cognitive status and APOE4 carriage. These results align with prior findings that periodic EEG measures, such as alpha peak frequency, decrease with age and cognitive impairment, while also highlighting the modulatory effects of genetic factors (Finley et al., 2023; Leroy et al., 2025).

#### 3.3.3 Spectral power ratio (SPR)

To investigate how the SPR varies with cognitive status, APOE4 genotype, and age, we analyzed SPR across all age groups. No significant main effects or other interactions were observed for this measure.

There was a significant two-way interaction between APOE4 and cognitive state for SPR [F_(2, 1152)_ = 5.303, *p* = 0.005] and a trend-level three-way interaction between APOE4, cognitive state, and age group [F_(4, 1152)_ = 1.655, *p* = 0.158]. Age-related contrasts in the naMCI APOE4 carriers showed heightened SPR in the <70 compared to the ≤75 [δ = 0.39, t(1152) = 2.807, Cohen’s *d* = 0.842, 95% CI: 0.06–0.73, *p* = 0.008) and >75 [δ = 0.59, t(1152) = 3.825, Cohen’s *d* = 1.264, 95% CI: 0.22–0.96, *p* < 0.001] groups. In CN, no significant differences in both APOE4 non-carriers and carriers were observed across all age groups.

In summary, SPR was particularly sensitive to the interaction between APOE4 status and MCI phenotype, with age-related differences evident only among naMCI APOE4 carriers. This suggests that SPR may serve as a useful potential marker for identifying at-risk subgroups within the MCI population, especially when considering genetic risk factors.

## 4 Discussion

This study aimed to investigate the combined effects of APOE ε4 and MCI on periodic and aperiodic EEG components using data from a portable two-channel device. The analysis focused on the main and interaction effects of APOE4 and MCI phenotypes (naMCI and aMCI) plus the CN reference group on resting-state EEG components, with particular emphasis on aperiodic activity. To provide a comprehensive understanding of these interactions, the effects of APOE4 were evaluated by adjusting for the demographic characteristics of age, sex, and years of education. Furthermore, the study examined how the interplay between APOE4, cognitive status, and age (<70, ≤75, and >75 years) influenced these EEG components while controlling for sex and years of education.

These analyses revealed significant interactions between APOE4 and cognitive states in the aperiodic EEG component (slope/exponent and offset) and the conventional spectral analysis measure of SPR. Among the APOE ε4 carriers, the aMCI individuals exhibited heightened aperiodic activity and a reduced SPR than the naMCI. However, no differences in the aperiodic component and SPR were observed in the APOE4 non-carriers among the cognitive states. For reference, APOE4 carriers with CN also showed lower SPR than naMCI individuals, further highlighting the distinct profile of naMCI within this genetic context (Figure 3).

Relying on the assumption that low frequency power is dominated by inhibitory currents (Brake et al., 2024), there is evidence from computational models and experiments linking aperiodic activity to an E/I balance of neuronal population (Gao et al., 2017), with a heightened exponent attributed to increased inhibition (Colombo et al., 2019; Lendner et al., 2020), and a reduced exponent attributed to increased excitation (Zsido et al., 2022). There is also growing evidence of alterations in neuronal E/I balance as a potential cause of neural network dysfunction in AD (Maestú et al., 2021; van Bueren et al., 2023; McKeon et al., 2024). Our findings present a mixed pattern: among APOE4 carriers, the aperiodic component is elevated in aMCI compared to naMCI, whereas the distribution in the aperiodic component is the same in non-carriers. The observed higher offsets and exponents in the aMCI group relative to the naMCI group, specifically among APOE4 carriers, align with expectations. This pattern likely reflects the influence of APOE ε4 on AD-related symptoms and may signify early pathways of AD pathology, which are more commonly associated with aMCI than naMCI (Ma et al., 2022). However, interpreting aperiodic EEG changes as direct markers of E/I balance remains debated (Maestú et al., 2021; van Bueren et al., 2023; McKeown et al., 2024), underscoring the need for multimodal validation and disease-specific calibration (Lendner et al., 2020; Waschke et al., 2021). Moreover, parameterization methods for separating periodic and aperiodic components such as specparam (Donoghue et al., 2020), or Irregular Resampling Auto-Spectral Analysis (IRASA) (Wen and Liu, 2016), may yield divergent estimates of the aperiodic exponent and offset, potentially impacting the reliability and comparability of findings across studies (Tröndle et al., 2022). As such, while our results highlight associations between APOE4 status, cognitive phenotype, and aperiodic EEG features, these findings should be interpreted with caution. Moreover, in APOE4 carriers, oscillatory differences could arise from gamma-band hyperactivity indicating local circuit hyperexcitability or disrupted long-range connectivity in alpha/beta bands (Canuet et al., 2012; Koelewijn et al., 2019). Also, much as studies such as (Choi et al., 2020) have demonstrated high conformity of the prefrontal lobe to all brain regions in the EEG slowing aspects, other brain areas are reported to show different dominant EEG characteristics as they are involved in different brain dynamics (Jeong, 2004). Thus, our findings may not capture whole-brain pathophysiology. Additionally, there are contrasting results in the context of MCI/AD research, for example, (Azami et al., 2023) did not find any differences in the aperiodic component between AD, MCI, and CN individuals, while studies such as (Chu et al., 2023) identified elevated aperiodic activity in AD-related pathology. These discrepancies could be due to differences in patient heterogeneity, EEG channel locations, analytic methods, or lack of genetic stratification. Our work tries to address this by analysis based on MCI phenotypes as well as genetic predisposition.

Next, the heightened SPR seen in naMCI compared to aMCI and CN in only APOE4 carriers could represent neural network dysfunction (Maestú et al., 2021), with a shift to higher excitation in the naMCI compared to the aMCI and CN APOE4 carriers. The SPR is a measure of the shift in power distribution from high to lower frequencies, with a higher SPR indicating more power in the high compared to low frequency neural activity and vice versa. For example, SPR has been reported to be lower in amyloid beta positive aMCI patients compared to the cognitively normal ones (Flores-Sandoval et al., 2023). Going by the above, the increased SPR could suggest higher power distribution in high-frequency neural activity in the naMCI compared to the aMCI and CN APOE4 carriers. This suggests that naMCI individuals who are APOE4 carriers may compensate for cognitive deficits, commonly associated with preclinical stages or neurodegeneration, by recruiting additional neural resources or exhibiting hyperactivation in specific brain regions (Scheller et al., 2014). Such mechanisms enable them to perform at a level comparable to their CN counterparts; otherwise would have shown more severe cognitive impairment. The interplay between the APOE4 and the cognitive states, particularly the MCI phenotypes in the aperiodic component seen above, underscores the importance of considering both genetic predisposition and MCI phenotypes in understanding early stages of cognitive decline. It further suggests that the presence or absence of the APOE ε4 allele may influence the neural and cognitive profiles of individuals with MCI, which could be important for early diagnosis and personalized treatment approaches for Alzheimer’s disease.

Next, our analyses of the interplay between APOE4 status, cognitive phenotype, and age group showed no interactions. Notably, age-related declines in SPR, and the periodic measures of peak_cf, peak_pw, and peak_bw were more pronounced in the MCI subtypes compared to CN. There were distinct patterns in naMCI, particularly among APOE4 carriers across the age groups. In contrast, in the aMCI, significant age-related differences were most evident among APOE4 non-carriers. CN participants showed fewer significant age-related differences across APOE4 groups, whereas most differences in periodic activity were in the naMCI and aMCI. These findings underscore the complex interplay between APOE4 genotype, aging, and cognitive impairment, and suggest that periodic EEG alterations may be particularly sensitive to disease stage and genetic risk within MCI populations.

These age group-based analyses reveal age and APOE4-dependent modulation of EEG peak frequencies, extending prior work on genetic and demographic influences in cognitive decline (Smailovic et al., 2021; Choi et al., 2023; Finley et al., 2023). The observed higher peak frequency measures in younger (<70 years) compared to the older (>75 years) CN non-carriers likely reflects age-related neural slowing, consistent with established patterns of alpha/beta peak frequency reduction in healthy aging (Flores-Sandoval et al., 2023; Smith et al., 2023; Choi et al., 2024). The significantly heightened peak frequencies in younger naMCI APOE4 carriers support the neural compensation hypothesis, where the ε4 allele drives early hyperactivation to counteract emerging pathology (Smailovic et al., 2021). The absence of significant effects in aMCI patients could suggest pathological saturation, where Alzheimer’s specific processes dominate EEG signatures (Polsinelli et al., 2023). Furthermore, sex emerged as a significant covariate in relationships between EEG measures, APOE4 status, cognitive states, and aging, reinforcing established sex differences in APOE4 effects. This could be related to estrogen’s neuroprotective properties or X-chromosome interactions, particularly given evidence of accelerated decline in female APOE4 carriers with early-onset Alzheimer’s disease (Polsinelli et al., 2023). The findings in our data show the 70–75-year window as a critical period of vulnerability for APOE4-related effects in MCI and AD, consistent with previous reports of age-dependent risk patterns (Wang J. et al., 2022; Eyamu et al., 2025). The distinct effects of APOE ε4 status, and age group on the aperiodic EEG activity and SPR suggest that both genetic and age-related factors could play a role in brain dynamics in MCI. Moreover, the >70 APOE4 carriers appear to show greater neural disruption.

This study shows distinct effects of APOE ε4 and cognitive states, particularly MCI on the often-overlooked non-oscillatory EEG activity, emphasizing the importance of considering both genetic factors and MCI phenotypes when studying the early stages of AD. It reveals that the presence or absence of the APOE ε4 allele may shape the neural aperiodic component profiles in individuals with MCI, potentially offering possible insights for early diagnosis and personalized treatment strategies.

While the results provide valuable insights, some limitations should be considered. First, there is limited generalizability of our findings as our participants are exclusively ethnically Korean. Ethnicity is known to impact genetic genotypes and, subsequently, AD occurrence (Lim et al., 2022; Miyashita et al., 2023). Again, on generalizability, our data were from a single center, thus, inherently carries a high risk of selection bias, so our study sample may not accurately represent the broader population of individuals with MCI or even cognitively normal older adults. Therefore, we recommend future studies to include multi-center recruitment and more diverse sampling strategies to enhance generalizability.

Secondly, our analysis did not consider other demographic factors such as premorbid IQ [used to make inferences about cognitive decline (Carlozzi et al., 2011)], social and economic status, smoking and drinking status, and a history of drug abuse. Our analysis also did not consider pertinent lifestyle factors, such as sleep quality and physical activity, as well as other additional factors, including current medication use (particularly psychotropic, neurological, or cardiovascular agents), which can independently influence EEG aperiodic components. Moreover, EEG was recorded at varying times of the day, a limitation that may contribute to variability related to circadian, ultradian rhythms, and participant fatigue (Croce et al., 2018; Lehnertz et al., 2021).

Furthermore, it would be beneficial to perform this analysis using EEG data collected from other brain regions to determine if these findings represent region-specific or whole-brain pathophysiology. We also acknowledge that the relatively high high-pass filter cutoff may have attenuated slow-frequency signals, which are integral to both aperiodic components and delta power (Frelih et al., 2024). This limitation could influence the estimation of these measures, including the SPR, and therefore should be considered when interpreting the results.

Finally, as this is a cross-sectional study, further research, particularly with longitudinal data, is needed to confirm and extend our findings.

In conclusion, this work revealed the interplay between APOE4 and the cognitive states, particularly MCI, in the aperiodic EEG component. Adjusted for age, sex, and years of education, there was a heightened aperiodic component in aMCI compared to the naMCI individuals observed in the APOE4 carriers, possibly indicative of the effects of APOE4 on AD-related symptoms. The distinct EEG spectral aperiodic components between aMCI and naMCI relative to CN in relation to APOE4 represent promising neurophysiological markers that may help identify individuals at elevated risk for cognitive decline or progression toward AD.

## Data Availability

The raw data used and/or analyzed in this article will be made available by the corresponding author(s) on reasonable request.
