# Supplementary material for: Aperiodic EEG signatures: unveiling the interplay between APOE ε4 and mild cognitive impairment subtypes
Source: Front Aging Neurosci. 2026 Jan 8;17:1675330. doi: 10.3389/fnagi.2025.1675330 (PMC12823931; doi:10.3389/fnagi.2025.1675330)
Supplement: Supplementary file 1 [file Data_Sheet_1.pdf]

Aperiodic EEG Signatures: Unveiling the Interplay between APOE ε4 and Mild Cognitive Impairment Subtypes

Joel Eyamu[1,2], Boncho Ku[1,2], Kahye Kim[1], Kun Ho Lee[3,4,5], and Jaeuk U. Kim[1,2*]

[1]Digital Health Research Division, Korea Institute of Oriental Medicine, Daejeon, South Korea
[2]KM Convergence Science, University of Science and Technology, Daejeon, South Korea
[3]Gwangju Alzheimer's Disease and Related Dementias (GARD) Cohort Research Center, Chosun University, Gwangju, South Korea
[4]Department of Biomedical Science, Chosun University, Gwangju, South Korea
[5]Dementia Research Group, Korea Brain Research Institute, Daegu, South Korea

*** Correspondence:**
Jaeuk U. Kim
jaeukkim@kiom.re.kr

1 Formula for periodic and aperiodic component estimation

The power spectral density (PSD), $P(f)$ for each frequency *f* is expressed as

$$P(f) = L(f) + \sum_n G_n(f) \quad (1),$$

where $P(f)$ is composed of the aperiodic component $L(f)$ and Gaussians $G_n(f)$.

The aperiodic components were parameterized using a Lorentzian function as;

$L(f) = b - \log(k + f^x)$ (2), where b is the broadband 'offset', k is the 'knee and x is the 'exponent' of the aperiodic fit. In addition, the periodic component was parameterized as a mixture of Gaussian distributions:

$$G_n = \alpha * \exp\left(\frac{-(f-c)^2}{2*\omega^2}\right)$$

(3), where α is the height of the peak over and above the aperiodic component (power), *c* is the center frequency of the peak (center frequency), ω is the width of the peak (bandwidth) and *f* is the set of frequency values (Wang *et al.*, 2022).

2 Formula for spectral power Ratio (SPR)

$$SPR = \frac{\alpha+\beta}{\delta+\theta} \quad (4),$$

where δ is delta band (3-4 Hz), θ is theta band (4-8 Hz), α is the alpha band (8-13 Hz), and β is the beta band (13-30 Hz) powers based on the conventional band-based spectral analysis approach.

3 Figures

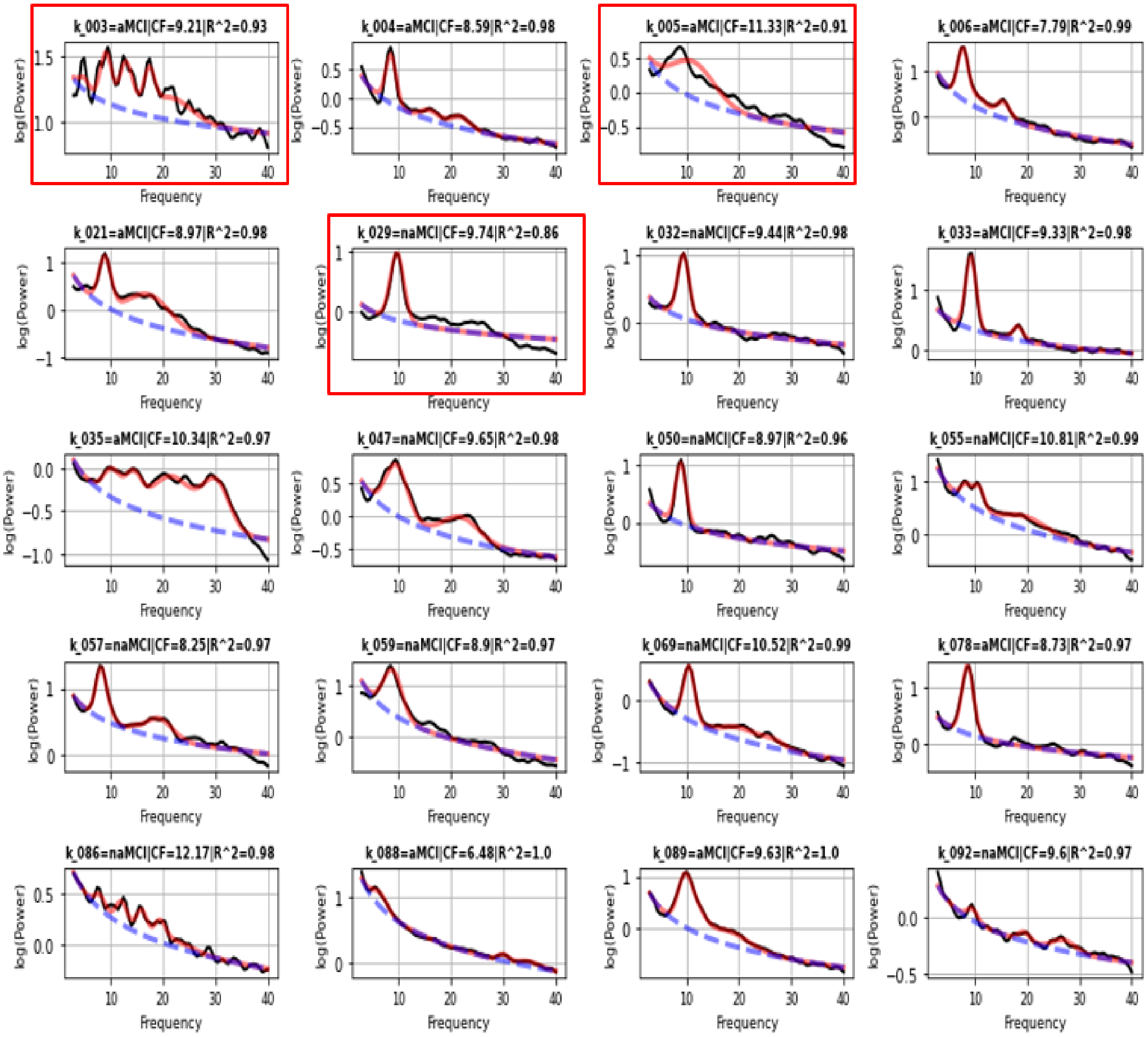

Figure S1: The original spectrum(black), full model (red) and aperiodic (dotted blue) fits for representative participants. CF: is the center frequency, R^2 is the coefficient of determination of the full model fit. naMCI, aMCI etc., are the respective cognitive states of the participants. Highlighted in Red are the poorly fit full models with $R^2 < 0.95$, so not considered in the ensuing analysis.

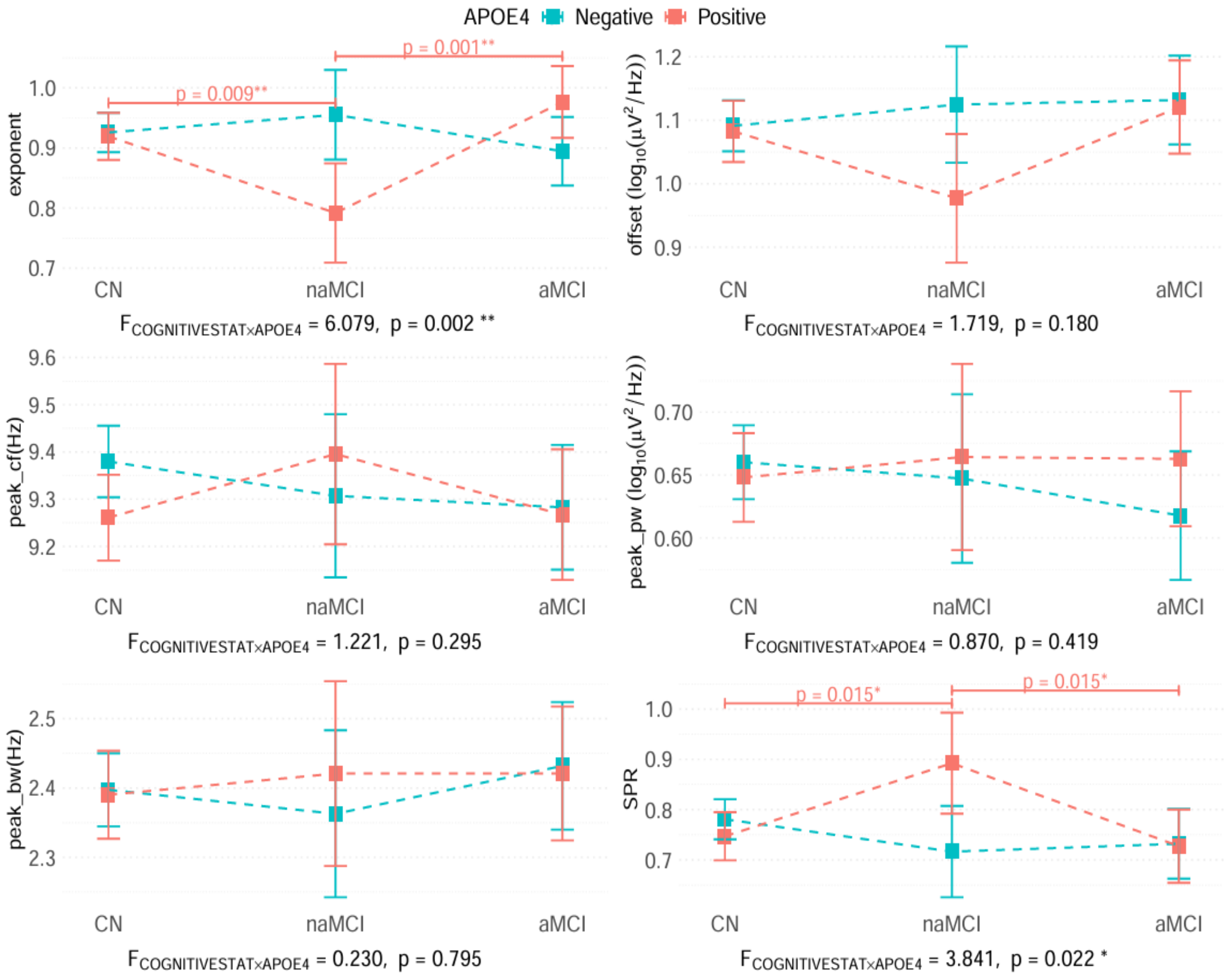

Figure S2: The interaction plot of the cognitive status and APOE4 in terms of the EEG spectral measures, including participants with R2 < 95 i.e., the 219, so overall participants analyzed here are CN = 883, naMCI=181 and aMCI = 327. This figure is related to Figure 3 (which had CN = 751, naMCI=142 and aMCI = 279) in the main text.

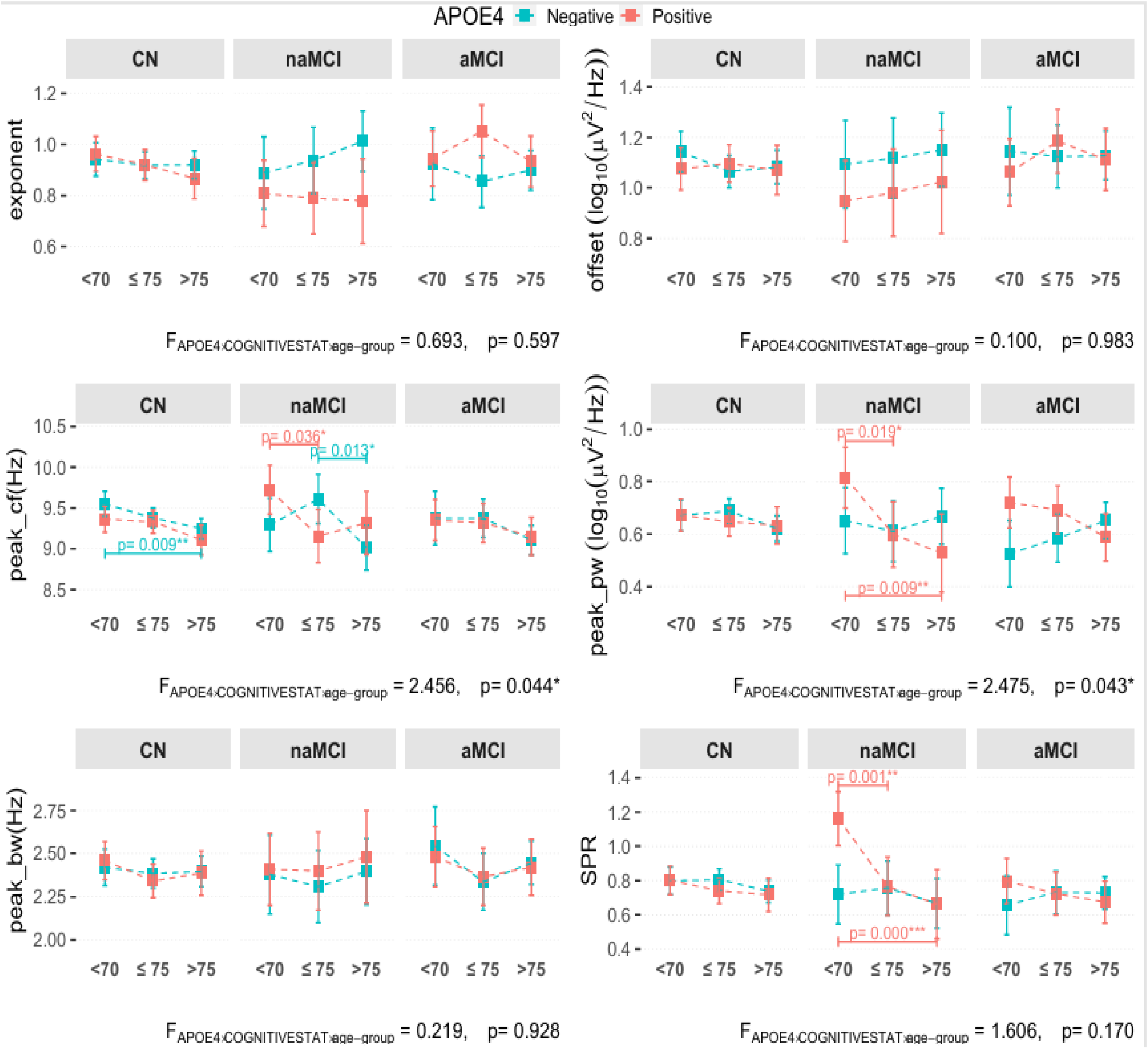

Figure S3: The interaction plot of the cognitive status, age group and APOE4 in terms of the EEG spectral measures, including participants with R2 < 95 i.e., the 219, so overall participants analyzed here are CN = 883, naMCI=181 and aMCI = 327. This figure is related to Figure 4 (which had CN = 751, naMCI=142 and aMCI = 279) in the main text.

4 Tables

**Table S1**: Post-hoc differences in demographic and neuropsychological test characteristics			
Characteristic	Comparison	Z	P.adj[1]
AGE	aMCI - CN	3.559	**0.001**
	aMCI - naMCI	2.120	0.068
	CN - naMCI	-0.338	0.735
SEX	CN : naMCI		0.580
	CN : aMCI		**0.002**
	naMCI : aMCI		**0.006**
MMSE	aMCI - CN	-8.551	**<0.001**
	aMCI - naMCI	-1.827	0.068
	CN - naMCI	4.493	**<0.001**
Attention	aMCI - CN	-7.135	**<0.001**
	aMCI - naMCI	1.386	0.166
	CN - naMCI	7.028	**<0.001**
Language	aMCI - CN	-9.538	**<0.001**
	aMCI - naMCI	1.543	0.123
	CN - naMCI	9.047	**<0.001**
Visuospatial	aMCI - CN	-6.526	**<0.001**
	aMCI - naMCI	-0.433	0.665
	CN - naMCI	4.513	**<0.001**
Memory	aMCI - CN	-20.138	**<0.001**
	aMCI - naMCI	-9.688	**<0.001**
	CN - naMCI	4.516	**<0.001**
Frontal	aMCI - CN	-9.652	**<0.001**
	aMCI - naMCI	-0.296	0.767
	CN - naMCI	7.061	**<0.001**

**Table S1**: Post-hoc differences in demographic and neuropsychological test characteristics			
Characteristic	Comparison	Z	P.adj[1]

Table S1: Z: Values for the Z test statistic for each comparison; [1]FDR adjusted p-values from Dunn Test for Kruskal Wallis multiple comparisons or Chi-squared test; The participant composition in SEX include; CN (Female= 417, Male = 334), naMCI (Female = 83, Male = 59), and aMCI (Female = 121, Male = 158).

Table S2: Key demographic and clinical variables including age, sex, cognitive status (CN/naMCI/aMCI), and APOE ε4 distribution.

**Characteristic**	**R Squared < 0.95**<br>N = 219[1]	**R Squared ≥ 0.95**<br>N = 1,172[1]	**T- statistic**[2]	**p-value**[2]
**COGNITIVESTAT**			5.295	0.071
CN	132 / 219 (60%)	751 / 1,172 (64%)		
naMCI	39 / 219 (18%)	142 / 1,172 (12%)		
aMCI	48 / 219 (22%)	279 / 1,172 (24%)		
**AGE**	72.84 (5.58)	72.67 (6.18)	0.412	0.7
**SEX**			11.295	**<0.001**
Female	143 / 219 (65%)	621 / 1,172 (53%)		
Male	76 / 219 (35%)	551 / 1,172 (47%)		
**EDUYR**	10.54 (4.54)	11.10 (4.35)	-1.695	0.091
**APOE4**			0.353	0.6
Negative	129 / 219 (59%)	665 / 1,172 (57%)		
Positive	90 / 219 (41%)	507 / 1,172 (43%)		

Table S2: [1]Mean (SD); n / N (%);[2]Pearson's Chi-squared test; Welch Two Sample t-test: The table shows the group differences between excluded participants due to poor specParam algorithm fit (R squared < 0.95) and those considered in the main analysis (R squared ≥ 0.95). Due to the differences in sex, further analysis was done to ascertain if including the former group did not change the overall trend of results (See Figures S2 and S3)

Table S3: Differences in demographic and neuropsychological characteristics in APOE4 groups in each cognitive state

	**CN: APOE ε4- Vs APOE ε4+**				**naMCI: APOE ε4- Vs APOE ε4+**				**aMCI: APOE ε4- Vs APOE ε4+**			
**Characteristic**	**Non-carriers, N = 438**[1]	**Carriers, N = 313**[1]	**T-statistic**	**p-value**[2]	**Non-carriers, N = 82**[1]	**Carriers, N = 60**[1]	**T-statistic**	**p-value**[2]	**Non-carriers, N = 145**[1]	**Carriers, N = 134**[1]	**T-statistic**	**p-value**[2]
**Demographic Characteristics**												
**AGE**	73.03 (6.22)	71.27 (5.64)	3.963	**<0.001**	73.82 (6.16)	70.87 (5.66)	2.914	**0.004**	74.93 (6.39)	72.45 (6.33)	3.257	**0.001**
**SEX**			0.072	0.8			1.120	0.3			0.883	0.3
Female	245 / 438 (56%)	172 / 313 (55%)			51 / 82 (62%)	32 / 60 (53%)			59 / 145 (41%)	62 / 134 (46%)		
Male	193 / 438 (44%)	141 / 313 (45%)			31 / 82 (38%)	28 / 60 (47%)			86 / 145 (59%)	72 / 134 (54%)		
**EDU YR**	11.06 (4.17)	11.07 (4.46)	-0.020	>0.9	11.35 (4.23)	11.34 (4.26)	0.008	>0.9	11.37 (4.41)	10.76 (4.72)	1.111	0.3
**Neuropsychological test scores**												
**MMSE**	27.87 (1.70)	27.76 (1.85)	0.880	0.4	26.80 (2.64)	26.85 (2.18)	-0.108	>0.9	26.59 (2.50)	26.02 (2.85)	1.781	0.076
**Attention**	9.68 (2.11)	9.74 (2.18)	-0.393	0.7	8.39 (2.05)	8.37 (2.11)	0.067	>0.9	8.65 (1.90)	8.60 (1.92)	0.224	0.8
**Language**	0.19 (0.29)	0.22 (0.24)	-1.882	0.060	-0.14 (0.50)	-0.07 (0.38)	-0.906	0.4	-0.11 (0.51)	-0.01 (0.41)	-1.821	0.070

Table S3: Differences in demographic and neuropsychological characteristics in APOE4 groups in each cognitive state

	**CN: APOE ε4- Vs APOE ε4+**				**naMCI: APOE ε4- Vs APOE ε4+**				**aMCI: APOE ε4- Vs APOE ε4+**			
**Characteristic**	**Non-carriers, N = 438[1]**	**Carriers, N = 313[1]**	**T-statistic**	**p-value[2]**	**Non-carriers, N = 82[1]**	**Carriers, N = 60[1]**	**T-statistic**	**p-value[2]**	**Non-carriers, N = 145[1]**	**Carriers, N = 134[1]**	**T-statistic**	**p-value[2]**
**Visuospatial**	0.50 (0.39)	0.51 (0.39)	-0.538	0.6	0.20 (0.71)	0.28 (0.70)	-0.668	0.5	0.17 (0.69)	0.18 (0.85)	-0.132	0.9
**Memory**	0.27 (0.57)	0.29 (0.58)	-0.454	0.6	-0.04 (0.55)	0.10 (0.47)	-1.532	0.13	-0.70 (0.47)	-0.71 (0.61)	0.225	0.8
**Frontal**	0.24 (0.56)	0.27 (0.55)	-0.737	0.5	-0.22 (0.65)	-0.10 (0.65)	-1.073	0.3	-0.18 (0.62)	-0.28 (0.77)	1.293	0.2

Table S3: [1]Mean (SD); n / N (%);[2]Two Sample t-test; Pearson's Chi-squared test; Welch Two Sample t-test:

The table shows the group differences between APOE statuses (carriers and non-carriers) across the cognitive states. First, in the CN, there were 438 non-carriers and 313 carriers. The non-carriers had 56% women and 44% men, while the carriers comprised of 55% women and 45% men. There was a statistically significant difference in age, non-carriers were older, with a mean age (standard deviation) of 73.03 (6.22) years compared to carriers 71.27 (5.64) years ($p < 0.001$). Next, in the naMCI, there were 82 non-carriers and 60 carriers. The non-carriers had 62% women and 38% men, while the carriers comprised of 53% women and 47% men. The non-carriers were older, with a mean age (standard deviation) of 73.82 (6.16) years compared to carriers 70.87 (5.66) years ($p = 0.004$). In the aMCI group, there were 145 non-carriers and 134 carriers. The non-carriers had 41% women and 59% men, while the carriers comprised of 46% women and 54% men. Just like in the CN and naMCI, the non-carriers were older, with a mean age (standard deviation) of 74.93 (6.39) years compared to carriers 72.45 (6.33) years ($p = 0.001$). No statistically significant differences were noted in sex, years of education, and any of the neuropsychological test scores between the APOE4 groups across all the cognitive states.

Table S4: The relationship between the APOE4 and Cognitive status with various EEG measures

		Unadjusted[1]			Adjusted[2]		
**EEG**	**Term**	**DF[1]**	**f-value[1]**	**p-value[1]**	**DF[2]**	**f-value[2]**	**p-value[2]**
**exponent**	APOE4	1	0.000	0.983	1	0.000	0.983
	COGNITIVESTAT	2	1.333	0.264	2	1.328	0.266
	APOE4:COGNITIVESTAT	2	4.878	**0.008**	2	4.868	**0.008**
	AGE				1	0.120	0.729
	SEX				1	0.030	0.864
	EDUYR				1	0.132	0.717
**offset**	APOE4	1	0.318	0.573	1	0.273	0.601
	COGNITIVESTAT	2	0.776	0.461	2	0.857	0.425
	APOE4:COGNITIVESTAT	2	2.941	0.053	2	2.612	0.074
	AGE				1	0.008	0.927
	SEX				1	11.745	**0.001**
	EDUYR				1	2.200	0.138
**peak_cf**	APOE4	1	0.915	0.339	1	2.586	0.108
	COGNITIVESTAT	2	1.327	0.266	2	0.372	0.689
	APOE4:COGNITIVESTAT	2	0.397	0.672	2	0.277	0.758
	AGE				1	30.474	**<0.001**
	SEX				1	8.497	**0.004**
	EDUYR				1	0.409	0.523
**peak_pw**	APOE4	1	0.000	0.987	1	0.060	0.807
	COGNITIVESTAT	2	0.697	0.498	2	0.859	0.424
	APOE4:COGNITIVESTAT	2	0.753	0.471	2	0.720	0.487
	AGE				1	3.941	**0.047**

Table S4: The relationship between the APOE4 and Cognitive status with various EEG measures

		**Unadjusted[1]**			**Adjusted[2]**		
**EEG**	**Term**	**DF[1]**	**f-value[1]**	**p-value[1]**	**DF[2]**	**f-value[2]**	**p-value[2]**
	SEX				1	6.269	**0.012**
	EDUYR				1	0.056	0.813
**peak_bw**	APOE4	1	0.477	0.490	1	0.520	0.471
	COGNITIVESTAT	2	0.085	0.919	2	0.006	0.994
	APOE4:COGNITIVESTAT	2	0.332	0.717	2	0.337	0.714
	AGE				1	0.268	0.605
	SEX				1	12.903	**<0.001**
	EDUYR				1	0.160	0.689
**SPR**	APOE4	1	0.519	0.472	1	1.270	0.260
	COGNITIVESTAT	2	1.461	0.233	2	0.993	0.371
	APOE4:COGNITIVESTAT	2	4.022	**0.018**	2	3.752	**0.024**
	AGE				1	11.619	**0.001**
	SEX				1	0.033	0.856
	EDUYR				1	0.768	0.381

[1]Model without adjustment; [2]Model with age, sex and years of education as covariates; DF = Degree of Freedom; p-values <0.05 are bolded

Table S4: The results were obtained from a generalized linear model (GLM) with an identity function. All the statistical measures were obtained from type III ANOVA of the GML models; APOE4:COGNITIVESTAT is the interaction between APOE4 and the cognitive status in each of the EEG measures. The residuals of the unadjusted and adjusted models were 1,166 and 1,163 respectively;

**Table S5:** The contrasts by cognitive states in EEG measures in the APOE4							
		Negative			Positive		
Feature	Contrast	δ (95% CI)	ES	p-value	δ (95% CI)	ES	p-value
exponent	CN - naMCI	-0.06 (-0.16, 0.05)	-0.156	0.295	0.10 (-0.02, 0.22)	0.278	0.074
	CN - aMCI	0.02 (-0.06, 0.11)	0.069	0.472	-0.05 (-0.14, 0.04)	-0.145	0.156
	naMCI - aMCI	0.08 (-0.04, 0.20)	0.225	0.295	-0.15 (-0.28, -0.02)	-0.424	**0.020**
offset	CN - naMCI	-0.05 (-0.17, 0.06)	-0.136	0.581	0.10 (-0.03, 0.23)	0.262	0.095
	CN - aMCI	-0.03 (-0.12, 0.06)	-0.083	0.581	-0.05 (-0.15, 0.05)	-0.127	0.220
	naMCI - aMCI	0.02 (-0.11, 0.15)	0.053	0.701	-0.15 (-0.30, -0.01)	-0.389	**0.038**
peak_cf	CN - naMCI	0.05 (-0.19, 0.29)	0.061	0.914	-0.06 (-0.34, 0.22)	-0.072	0.803
	CN - aMCI	0.06 (-0.13, 0.25)	0.076	0.914	0.02 (-0.18, 0.23)	0.026	0.803
	naMCI - aMCI	0.01 (-0.26, 0.29)	0.015	0.914	0.08 (-0.23, 0.39)	0.100	0.803
peak_pw	CN - naMCI	-0.02 (-0.12, 0.08)	-0.048	0.692	-0.06 (-0.17, 0.06)	-0.168	0.645
	CN - aMCI	0.04 (-0.04, 0.12)	0.111	0.381	-0.02 (-0.10, 0.07)	-0.048	0.645
	naMCI - aMCI	0.05 (-0.06, 0.17)	0.158	0.381	0.04 (-0.09, 0.17)	0.120	0.645
peak_bw	CN - naMCI	0.01 (-0.16, 0.17)	0.012	0.972	-0.08 (-0.27, 0.12)	-0.130	0.686
	CN - aMCI	-0.00 (-0.14, 0.13)	-0.003	0.972	-0.04 (-0.18, 0.10)	-0.067	0.686
	naMCI - aMCI	-0.01 (-0.20, 0.18)	-0.015	0.972	0.04 (-0.18, 0.25)	0.063	0.686
SPR	CN - naMCI	0.06 (-0.08, 0.19)	0.125	0.448	-0.18 (-0.34, -0.02)	-0.382	**0.016**
	CN - aMCI	0.05 (-0.06, 0.16)	0.110	0.448	0.01 (-0.11, 0.12)	0.017	0.871

**Table S5:** The contrasts by cognitive states in EEG measures in the APOE4							
		Negative			Positive		
Feature	Contrast	δ (95% CI)	ES	p-value	δ (95% CI)	ES	p-value
	naMCI - aMCI	-0.01 (-0.16, 0.15)	-0.016	0.910	0.19 (0.01, 0.36)	0.400	**0.016**

Table S5: Models adjusted for age, sex and years of education; p-values for the contrasts are adjusted using False discovery rate (FDR) method; p-values <0.05 are bolded; Pairwise Cohen's D effect sizes (ES) were calculated as the difference in model-adjusted means divided by the model's residual standard deviation.

Table S6: The relationship between the APOE4 and Age group with various spectral EEG measures

Feature	Term	DF	F-value	P-value
exponent	APOE4	1	0.027	0.870
	age_group	2	0.247	0.782
	COGNITIVESTAT	2	0.069	0.934
	SEX	1	0.014	0.907
	EDUYR	1	0.110	0.740
	APOE4:age_group	2	0.048	0.953
	APOE4:COGNITIVESTAT	2	0.869	0.420
	age_group:COGNITIVESTAT	4	0.290	0.885
	APOE4:age_group:COGNITIVESTAT	4	0.866	0.483
offset	APOE4	1	1.713	0.191
	age_group	2	0.646	0.524
	COGNITIVESTAT	2	0.415	0.661
	SEX	1	12.327	**<0.001**
	EDUYR	1	1.846	0.175
	APOE4:age_group	2	0.879	0.416
	APOE4:COGNITIVESTAT	2	1.145	0.319
	age_group:COGNITIVESTAT	4	0.134	0.970
	APOE4:age_group:COGNITIVESTAT	4	0.280	0.891
peak_cf	APOE4	1	1.763	0.184
	age_group	2	4.248	**0.015**
	COGNITIVESTAT	2	0.620	0.538
	SEX	1	9.695	**0.002**
	EDUYR	1	0.838	0.360
	APOE4:age_group	2	0.441	0.644
	APOE4:COGNITIVESTAT	2	1.620	0.198
	age_group:COGNITIVESTAT	4	1.123	0.344

**Table S6**: The relationship between the APOE4 and Age group with various spectral EEG measures				
Feature	Term	DF	F-value	P-value
	APOE4:age_group:COGNITIVESTAT	4	2.170	0.070
peak_pw	APOE4	1	0.060	0.807
	age_group	2	2.735	0.065
	COGNITIVESTAT	2	2.443	0.087
	SEX	1	5.730	**0.017**
	EDUYR	1	0.095	0.758
	APOE4:age_group	2	0.464	0.629
	APOE4:COGNITIVESTAT	2	2.395	0.092
	age_group:COGNITIVESTAT	4	1.965	0.098
	APOE4:age_group:COGNITIVESTAT	4	2.170	0.070
peak_bw	APOE4	1	0.005	0.946
	age_group	2	0.086	0.918
	COGNITIVESTAT	2	1.124	0.325
	SEX	1	12.072	**0.001**
	EDUYR	1	0.317	0.574
	APOE4:age_group	2	0.360	0.698
	APOE4:COGNITIVESTAT	2	0.450	0.638
	age_group:COGNITIVESTAT	4	1.309	0.265
	APOE4:age_group:COGNITIVESTAT	4	0.954	0.432
SPR	APOE4	1	0.023	0.880
	age_group	2	1.497	0.224
	COGNITIVESTAT	2	1.228	0.293
	SEX	1	0.117	0.733
	EDUYR	1	1.172	0.279
	APOE4:age_group	2	0.204	0.816
	APOE4:COGNITIVESTAT	2	5.303	**0.005**
	age_group:COGNITIVESTAT	4	0.423	0.792
	APOE4:age_group:COGNITIVESTAT	4	1.655	0.158

Table S6: The results were obtained from a GLM with an identity function. All the statistical measures were obtained from type III ANOVA of the GML models; The residuals of the models were 1,152; significant p-values are bolded; DF: Degrees of freedom.

Table S7: Contracts between the Age groups considering APOE4 and cognitive state in the various spectral EEG measures

		CN		naMCI		aMCI	
Feature	Contrast	Negative	Positive	Negative	Positive	Negative	Positive
		δ(95% CI)[ES,p]	δ(95% CI)[ES,p]	δ(95% CI)[ES,p]	δ(95% CI)[ES,p]	δ(95% CI)[ES,p]	δ(95% CI)[ES,p]
exponent	<70 vs ≤ 75	0.02 (-0.09, 0.13)[0.056, 0.769]	0.04 (-0.07, 0.15)[0.111, 0.622]	0.01 (-0.24, 0.26)[0.030,0.917]	-0.00 (-0.26, 0.25)[-0.010,0.972]	0.05 (-0.17, 0.27)[0.143, 0.864]	-0.17 (-0.35, 0.01)[-0.482, 0.076]
	<70 vs >75	0.03 (-0.08, 0.14)[0.088,0.769]	0.04 (-0.09, 0.18)[0.126, 0.622]	-0.06 (-0.29, 0.18)[-0.159,0.852]	-0.04 (-0.32, 0.24)[-0.010,0.972]	0.01 (-0.19, 0.21)[0.036, 0.878]	-0.06 (-0.24, 0.11)[-0.180,0.393]
	≤75 vs >75	0.01 (-0.08, 0.11)[0.033,0.769]	0.01 (-0.12, 0.13)[0.016, 0.917]	-0.07 (-0.29, 0.15)[-0.189,0.852]	-0.04 (-0.34, 0.27)[-0.109,0.972]	-0.04 (-0.20, 0.12)[-0.107,0.864]	0.11 (-0.07, 0.29)[0.302, 0.232]
offset	<70 vs ≤ 75	0.03 (-0.09, 0.15)[0.008,0.550]	-0.03 (-0.15, 0.10)[-0.007,0.743]	0.09 (-0.18, 0.37)[0.025,0.686]	-0.02 (-0.30, 0.26)[-0.004,0.887]	0.01 (-0.23, 0.25)[0.002, 1.000]	-0.19 (-0.39, 0.01)[-0.048,0.074]
	<70 vs >75	0.06 (-0.06, 0.17)[0.014,0.550]	-0.05 (-0.19, 0.10)[-0.012,0.743]	0.08 (-0.18, 0.34)[0.021,0.686]	-0.08 (-0.39, 0.23)[-0.021,0.887]	0.01 (-0.21, 0.23)[0.002, 1.000]	-0.13 (-0.33, 0.06)[-0.034,0.155]
	≤75 vs >75	0.03 (-0.08, 0.13)[0.007,0.550]	-0.02 (-0.16, 0.12)[-0.005,0.743]	-0.01 (-0.25, 0.23)[-0.003,0.915]	-0.06 (-0.40, 0.27)[-0.017,0.887]	-0.00 (-0.18, 0.18)[0.000, 1.000]	0.06 (-0.14, 0.25)[0.014, 0.504]
peak_cf	<70 vs ≤ 75	0.18 (-0.07, 0.43)[0.216,0.126]	0.06 (-0.20, 0.31)[0.069, 0.595]	-0.23 (-0.81, 0.34)[-0.282,0.334]	**0.64 (0.05, 1.23)[0.775,0.030]**	0.02 (-0.49, 0.52)[0.021, 0.936]	0.09 (-0.34, 0.51)[0.106, 0.624]
	<70 vs >75	**0.30 (0.05, 0.55)[0.366,0.011]**	0.30 (-0.01, 0.60)[0.360, 0.061]	0.27 (-0.28, 0.83)[0.333,0.334]	0.36 (-0.30, 1.01)[0.433,0.286]	0.38 (-0.09, 0.84)[0.454, 0.083]	0.29 (-0.12, 0.71)[0.347, 0.270]
	≤75 vs >75	0.12 (-0.10, 0.34)[0.151,0.174]	0.24 (-0.05, 0.53)[0.290, 0.075]	0.51 (-0.00, 1.02)[0.614,0.053]	-0.28 (-0.98, 0.42)[-0.342,0.333]	0.36 (-0.02, 0.74)[0.433, 0.069]	0.21 (-0.21, 0.63)[0.251, 0.356]
peak_pw	<70 vs ≤ 75	0.00 (-0.10, 0.10)[0.004,0.974]	0.02 (-0.08, 0.13)[0.072, 0.713]	0.07 (-0.17, 0.30)[0.190,0.776]	**0.22 (-0.02, 0.47)[0.657,0.043]**	-0.06 (-0.27, 0.15)[-0.187,0.464]	-0.03 (-0.21, 0.15)[-0.091,0.670]
	<70 vs >75	0.08 (-0.02, 0.18)[0.236,0.091]	0.04 (-0.08, 0.17)[0.126, 0.713]	0.03 (-0.20, 0.26)[0.008,0.776]	**0.32 (0.04, 0.59)[0.923,0.016]**	-0.13 (-0.313, 0.06)[-0.384,0.31]	0.09 (-0.08, 0.26)[0.270, 0.298]
	≤75 vs >75	0.08 (-0.01, 0.17)[0.232,0.091]	0.02 (-0.10, 0.14)[0.055, 0.713]	-0.04 (-0.25, 0.17)[-0.111,0.776]	0.09 (-0.20, 0.38)[0.266,0.453]	-0.07 (-0.22, 0.09)[-0.197,0.453]	0.12 (-0.05, 0.30)[0.362, 0.266]

Table S7: Contracts between the Age groups considering APOE4 and cognitive state in the various spectral EEG measures

		CN		naMCI		aMCI	
Feature	Contrast	Negative	Positive	Negative	Positive	Negative	Positive
		δ(95% CI)[ES,p]	δ(95% CI)[ES,p]	δ(95% CI)[ES,p]	δ(95% CI)[ES,p]	δ(95% CI)[ES,p]	δ(95% CI)[ES,p]
peak_bw	<70 vs ≤ 75	0.03 (-0.15, 0.20)[0.048,0.918]	0.11 (-0.08, 0.29)[0.182, 0.425]	0.14 (-0.26, 0.55)[0.243,0.607]	0.00 (-0.41, 0.42)[0.006,0.983]	**0.38 (0.02, 0.73)[0.651, 0.033]**	0.08 (-0.22, 0.38)[0.138, 0.869]
	<70 vs >75	0.01 (-0.17, 0.18)[0.013,0.918]	0.01 (-0.20, 0.23)[0.023, 0.884]	0.15 (-0.24, 0.53)[0.254,0.607]	-0.06 (-0.52, 0.40)[-0.108,0.983]	0.17 (-0.16, 0.50)[0.297, 0.209]	0.06 (-0.23, 0.35)[0.103, 0.869]
	≤75 vs >75	-0.02 (-0.17, 0.13)[-0.035,0.918]	-0.09 (-0.30, 0.11)[-0.159,0.425]	0.01 (-0.35, 0.37)[0.011,0.965]	-0.07 (-0.56, 0.42)[-0.115,0.983]	-0.20 (-0.47, 0.06)[-0.354,0.095]	-0.02 (-0.32, 0.27)[-0.035,0.869]
SPR	<70 vs ≤ 75	0.02 (-0.12, 0.16)[0.046,0.713]	0.07 (-0.07, 0.22)[0.160, 0.327]	-0.04 (-0.37, 0.28)[-0.092,0.752]	**0.39 (0.06, 0.73)[0.842,0.008]**	-0.04 (-0.33, 0.24)[-0.092, 0.927]	0.02 (-0.22, 0.26)[0.034, 0.876]
	<70 vs >75	0.09 (-0.05, 0.23)[0.197,0.261]	0.12 (-0.05, 0.29)[0.256, 0.294]	0.05 (-0.26, 0.36)[0.113,0.752]	**0.59 (0.22, 0.96)[1.264,<0.001]**	-0.05 (-0.31, 0.21)[-0.110,0.927]	0.13 (-0.10, 0.37)[0.286, 0.351]
	≤75 vs >75	0.07 (-0.05, 0.19)[0.151,0.261]	0.04 (-0.12, 0.21)[0.096, 0.516]	0.10 (-0.19, 0.38))[0.205,0.752]	-0.04 (-0.33, 0.24)[0.422,0.233]	-0.01 (-0.22, 0.20)[-0.017,0.927]	0.12 (-0.12, 0.35)[0.253, 0.351]

Table S7: Generalized Linear Model (GLM) contrast mean differences estimates (δ) with 95% confidence intervals (CI) and [Cohen's D effect-size, p-values] comparing EEG measures on age groups across the cognitive states of CN, naMCI and aMCI. p-values for the contrasts are adjusted using False discovery rate (FDR) method; Pairwise effect sizes (ES) were calculated as the difference in model-adjusted means divided by the model's residual standard deviation; Significant associations ($p < 0.05$) are bolded. The model was adjusted for sex and years of education.

5 Bibliography

Wang, Z. *et al.* (2022) "Separating the aperiodic and periodic components of neural activity in Parkinson's disease," *European Journal of Neuroscience*, 56(6), pp. 4889–4900. Available at: https://doi.org/10.1111/ejn.15774.
